# La maladie de Fanconi: à propos d'une nouvelle observation

**DOI:** 10.11604/pamj.2015.20.92.6116

**Published:** 2015-02-02

**Authors:** Anass Es-Seddiki, Anass Ayyad, Sahar Messouadi, Rim Amrani

**Affiliations:** 1 Service de Pédiatrie, CHU Mohammed VI, Oujda, Maroc

**Keywords:** Pancytopénie, malformation, duplication du pouce, petite taille, greffe de moelle, Pancytopenia, malformation, duplication of the thumb, small size, bone marrow transplantation

## Abstract

La maladie de Fanconi ou l'anémie de Fanconi (AF) est une maladie génétique rare à transmission autosomique récessive. Elle est marquée par une hétérogénéité phénotypique. Certains symptômes et notamment la triade classique faite d'une petite taille, d'un syndrome malformatif varié et parfois discret et d'une insuffisance médullaire d'apparition précoce, doivent faire évoquer le diagnostic. Nous rapportons le cas d'un enfant âgé de sept ans, suivi et traité pour une luxation congénitale des hanches, qui présentait une pancytopénie avec à l'examen clinique on note un faciès dysmorphique triangulaire, une duplication du pouce droit, une surélévation de l’épaule gauche et un retard staturo-pondéral.

## Introduction

**Figure 1:**
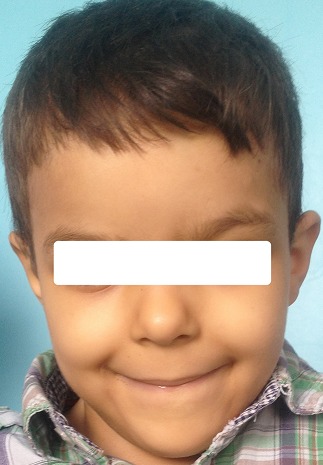
Face du patient avec faciès triangulaire et petit menton

**Figure 2:**
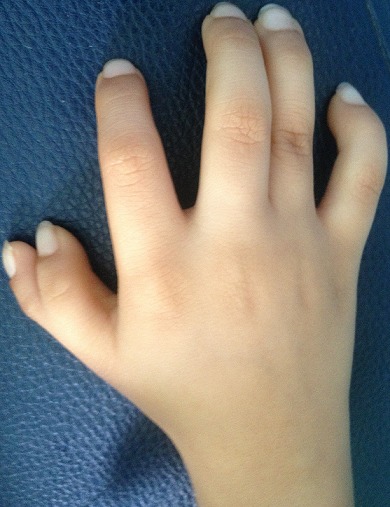
Main gauche montrant un pouce supplémentaire

La maladie de Fanconi ou l'anémie de Fanconi (AF) est un syndrome génétique humain rare à hérédité récessive, caractérisé par un phénotype extrêmement complexe et hétérogène [1–3]. Décrite par le pédiatre suisse Guido Fanconi pour la première fois en 1927; qui a publié un rapport sur une fratrie dont les membres présentaient une grave anémie aplasique associée à plusieurs malformations congénitales [1, 2]. Cette pathologie a été appelée l'anémie de Fanconi quelques années plus tard suite à la description d'autres cas similaires à ceux décrits par le Dr Fanconi. La principale manifestation clinique de l'AF est la présence d'une cytopénie pouvant toucher tous les éléments cellulaires du sang (pancytopénie) [1, 2]. Cette anomalie périphérique corrèle avec la présence d'une moelle hypoplasique (se déclarant au cours de la première décennie de vie). Les patients AF peuvent présenter plusieurs malformations [1–3]. Nous rapportons le cas d'un enfant âgé de sept ans, suivi et traité pour une luxation congénitale des hanches, qui a consulté pour une anémie sévère, dont l'examen clinique a retrouvé des signes en faveur de l'anémie de Fanconi: un faciès dysmorphique triangulaire (Figure 1), une duplication du pouce droit (Figure 2), une surélévation de l’épaule gauche et un retard staturo-pondéral.

## Patient et observation

**Figure 3:**
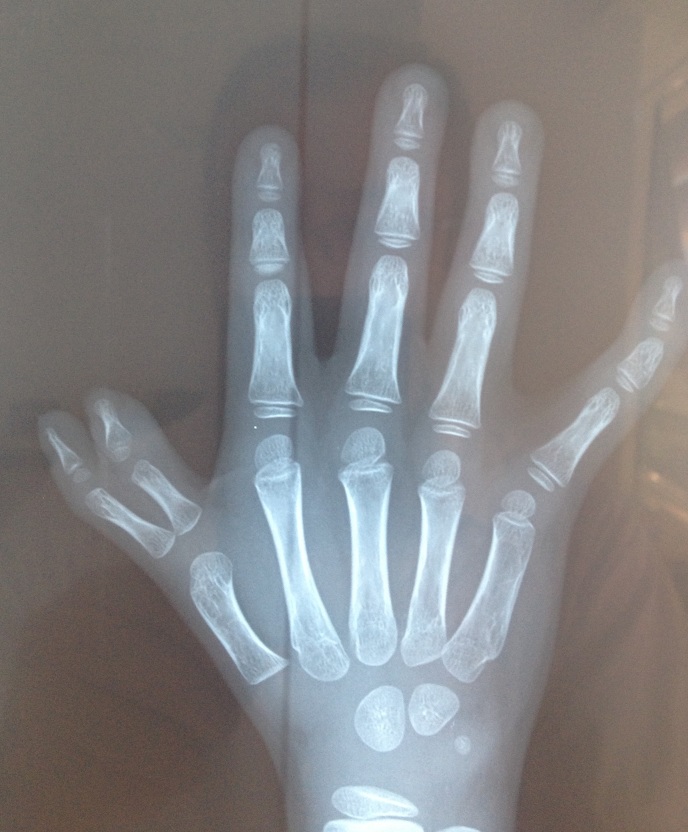
Radiographie de la main droite montrant une duplication du pouce type 4 selon la classification de Wassel

**Figure 4:**
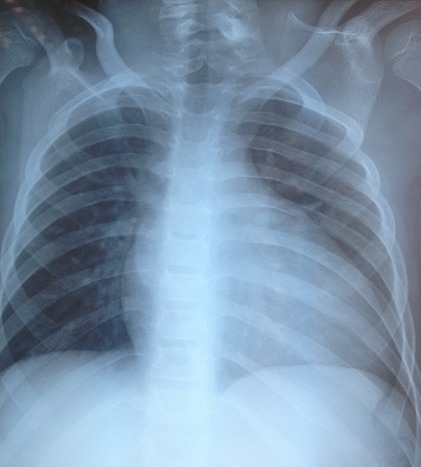
Radiographie du thorax face montrant la surélévation de l’épaule gauche

Il s'agit d'un enfant de sexe masculin, âgé de 07 ans, sans notion de consanguinité avec comme antécédent une luxation congénitale des hanches traitée à l’âge de 2 ans qui présente depuis un mois une anémie sévère. L'examen clinique à l'admission a retrouvé un poids à 17 kg (-2 D.S.), une taille à 115 cm (-1 D.S.), une tension artérielle correcte. On note aussi un faciès dysmorphique triangulaire, une petite taille, une duplication du pouce droit et une surélévation de l’épaule gauche. L'hémogramme a objectivé une pancytopénie avec une anémie macrocytaire; un taux d'hémoglobine à 3.9 g/dl, un volume globulaire moyen à 125.3 fl, un taux de leucocytes à 3960/mm^3^, des plaquettes à 20000/mm^3^ et un taux de réticulocytes à 80000 éléments/mm^3^ avec une ferritinémie à 120 ng/ml. Le dosage de la vitamine B12 et de l'acide folique a été normal. L'ionogramme sanguin a été sans anomalies y compris la fonction rénale et le taux des transaminases. Les anticorps anti transglutaminase IgG ont été normaux ainsi que le bilan thyroïdien. Le myélogramme a été en faveur d'une hypoplasie médullaire. L’échographie abdominale a été normale, la radiographie de la main droite a montré une duplication du pouce type 4 selon la classification de Wassel et un âge osseux estimé à 3 ans et demi selon l'Atlas de Greulich et Pyle (Figure 3) et la radiographie du thorax face a montré la surélévation de l’épaule gauche (Figure 4).

## Discussion

L'anémie de Fanconi est une maladie autosomique récessive rare. Elle se caractérise par une aplasie médullaire d'apparition progressive, des malformations congénitales et une prédisposition à développer des tumeurs malignes [1–3]. Le phénotype de l'anémie de Fanconi est très hétérogène, certains patients ayant des anomalies morphologiques importantes et une pancytopénie de début précoce, d'autres, au contraire n'ayant que peu ou pas d'anomalies morphologiques et ne développant que tardivement une pancytopénie [1–3]. On estime à un millier le nombre de patients atteints dans le monde [1]. La prévalence en France de l'AF est estimée à 1/300 000 naissances avec une fréquence de l'hétérozygotie estimée à 1/300 dans la population générale. L'AF est observée dans tous les groupes ethniques, avec une fréquence plus élevée chez les juifs ashkénazes et les afrikaners [1]. En Tunisie selon le registre tunisien de l'AF « TFAR » 142 cas sont répertoriés en 2012 [4]. L'AF appartient à un groupe de pathologies appelées « maladies cassantes », caractérisées par la présence de cassures chromosomiques, spontanées ou induites par l'exposition à différents agents susceptibles d'endommager l'ADN (agents antimitotiques, une instabilité génétique et une prédisposition au développement de cancers). Ces maladies regroupent, outre l'AF, l'ataxie télangiectasie, le syndrome de Bloom, et le syndrome de Nijmegen [1].

Les malformations squelettiques observées au cours de l'AF sont: les anomalies de la colonne radiale qui sont les plus fréquentes (environ un tiers des patients) et peuvent être uni ou bilatérales: pouce absent, hypoplasique, bifide, dupliqué ou tri phalange; hypoplasie de l’éminence thénar, absence du 1^er^ métacarpien; radius absent ou hypoplasique ou autre anomalie squelettique de la main. On observe également des malformations des membres inferieurs à type de syndactylie, des malformations des orteils, une luxation congénitale des hanches. Les anomalies du rachis également décrites sont la spina bifida, la scoliose, une hemivertèbre et l'aplasie coccygienne [1, 2, 5]. Des anomalies cutanées sont présentes chez 40% des patients, associant des taches café au lait ou des taches achromiques [1–3]. Des malformations rénales ou urinaires sont présentes chez environ 20% des patients. Il s'agit de reins uniques, en fer à cheval, ectopiques ou pelviens, hypoplasiques, dysplasiques ou encore d'une urétéro-hydronéphrose [1, 2]. Des anomalies oto-rhino-laryngologiques (ORL) touchent 10% des patients: malformation de l'oreille externe (malformation du pavillon de l'oreille, oreilles implantées basses, dysplasie ou atrésie du conduit auditif externe), ou surdité [1, 2]. Les anomalies du tube digestif concernent environ 7% des patients (l'atrésie de l’œsophage, avec ou sans fistule trachéo-œsophagienne, l'atrésie duodénale, l'imperforation anale et l'anus ectopique) [1, 2]. Enfin d'autres anomalies organiques peuvent être rencontrées: anomalies génitales présentes chez 25% des garçons (cryptorchidie, hypoplasie testiculaire, anorchidie, hypospadias, micro pénis), plus rares chez les filles (utérus bicorne, malposition utérine, hypoplasie ovarienne) [1, 2]. L'atteinte hématologique est le plus souvent absente à la naissance, l'hémogramme étant normal à ce stade. Progressivement se développe une anémie macrocytaire, suivie d'une thrombopénie et d'une neutropénie. L’âge médian d'apparition de la pancytopénie se situe vers 7 ans. Il existe un risque d’évolution vers un syndrome myélodysplasique ou une leucémie aigue [1–4, 6]. 15 gènes ont été identifiés. La mutation d'un de ces gènes est suffisante pour entrainer une AF. Le gène le plus souvent impliqué est FANCA(en 16q24.3) dans 60 à 70% des cas [1, 2]. L'insuffisance médullaire observée dans les 3/4 des cas dans la première décennie, doit faire l'objet d'une surveillance régulière. Un hémogramme doit être réalisé tous les 3 à 4 mois chez les patients stables ou sans anomalie clonale, et un myélogramme doit être réalisé chaque année à la recherche d'anomalies cytologiques ou caryotypiques associées aux myélodysplasies [1, 2, 7].

Pour les patients dont l'insuffisance médullaire s'aggrave ou chez qui une anomalie clonale apparaît, un traitement radical par greffe de cellules souches hématopoïétiques (CSH) doit être envisagé [1, 2]. Dans l'attente, les traitements symptomatiques doivent être envisagés avec modération. Le seuil transfusionnel en culots globulaires est fixé à 7 ou 8 g/dl d'hémoglobine avec des signes cliniques (ces transfusions doivent être limitées au maximum pour éviter toute allo-immunisation en pré greffe) [7]. Une surveillance de la surcharge en fer s'impose par le dosage de la ferritine, voire la réalisation d'une imagerie par résonance magnétique cardiaque ou hépatique pour démarrer un traitement par chélateur en fer [1–3]. L'utilisation de facteurs de croissance hématopoïétiques (G-CSF) doit être réservée aux patients neutropéniques dans les contextes d'infections sévères, l'usage de facteurs de croissance dans ce type de pathologie pouvant favoriser l’évolution clonale [1, 7]. L'administration d'androgènes permet une élévation du taux d'hémoglobine chez 50% des patients [1]. Pour notre patient, le diagnostic de l'AF a été posé devant l'association de pancytopénie, du pouce supplémentaire et des malformations associées notamment le faciès triangulaire, la luxation congénitale des hanches et la surélévation de l’épaule; malformation qui n'a jamais été décrite jusque là dans la littérature. Le diagnostique de l'AF a été confirmé par l’étude cytogénétique. Le traitement proposé pour notre cas a fait appel à une transfusion par des culots globulaires (phénotypé et déleucocytés) et les androgènes en attendant l'allogreffe de CSH.

## Conclusion

L'AF est une maladie génétique rare dont les symptômes débutent dans la première décade de vie. Il est nécessaire que les pédiatres évoquent le diagnostic devant certains signes isolés ou associés à une atteinte hématologique. La physiopathologie de la maladie a été bien décrite avec 15 gènes impliqués. Les patients atteints d'AF doivent bénéficier d'une prise en charge pluridisciplinaire en raison de la diversité des manifestations de la maladie, mais surtout d'une surveillance rapprochée vu le risque néoplasique. Le seul traitement curatif actuel de l'atteinte hématologique reste l'allogreffe de CSH.
